# Inducing Effect of Dihydroartemisinic Acid in the Biosynthesis of Artemisinins with Cultured Cells of *Artemisia annua* by Enhancing the Expression of Genes

**DOI:** 10.1155/2014/293190

**Published:** 2014-07-17

**Authors:** Jianhua Zhu, Jiazeng Yang, Zihan Zeng, Wenjin Zhang, Liyan Song, Wei Wen, Rongmin Yu

**Affiliations:** ^1^Biotechnological Institute of Chinese Materia Medica, Jinan University, Guangzhou 510632, China; ^2^College of Pharmacy, Jinan University, Guangzhou 510632, China

## Abstract

Artemisinin has been used in the production of “artemisinin combination therapies” for the treatment of malaria. Feeding of precursors has been proven to be one of the most effective methods to enhance artemisinin production in plant cultured cells. At the current paper, the biosynthesis of artemisinin (ART) and its four analogs from dihydroartemisinic acid (DHAA) in suspension-cultured cells of *Artemisia annua* were investigated. ARTs were detected by HPLC/GC-MS and isolated by various chromatography methods. The structures of four DHAA metabolites, namely, dihydro-epi-deoxyarteannuin B, arteannuin I, arteannuin K, and 3-*β*-hydroxy-dihydro-epi-deoxyarteannuin B, were elucidated by physicochemical and spectroscopic analyses. The correlation between gene expression and ART content was investigated. The results of RT-PCR showed that DHAA could up-regulate expression of amorpha-4,11-diene synthase gene (ADS), amorpha-4,11-diene C-12 oxidase gene (CYP71AV1), and farnesyl diphosphate synthase gene (FPS) (3.19-, 7.21-, and 2.04-fold higher than those of control group, resp.), which indicated that biosynthesis processes from DHAA to ART were enzyme-mediated.

## 1. Introduction

Malaria is one of the most prevalent and devastating parasitic diseases worldwide, with 350 to 500 million febrile episodes observed yearly in African children alone and more than 1 million malaria-related deaths each year [1]. Artemisinin (ART) is a drug obtained from the plant* Artemisia annua* that has been recently recommended by the WHO in combination with other antimalarial drugs to treat drug-resistant* Plasmodium falciparum* strains, cerebral malaria, and malaria in children [2]. Plant cultivation is one of the major strategies for the production of ART [3]. However, plant cultivation is time consuming (needs several months), affected by climate and insect. In addition, the concentration of ART in* A. annua* is relatively low and unstable [4, 5]. Attempts to produce the precursor of ART in engineered yeast have succeeded and ART could also be obtained by chemical synthesis. But no ART is directly produced in any engineered yeast. Bioengineered target organisms have been established attempting to produce ART but with low concentration [6–8]. Therefore, a considerable interest in recent years has been focused on understanding the natural biosynthetic pathways of ART, the enzymes involved, and the underlying genetic expressions [5].

**Figure 1:**
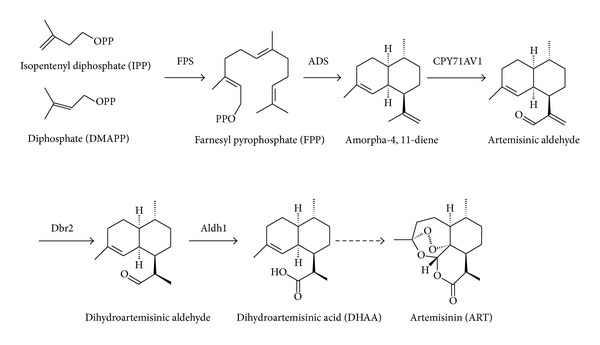
Biosynthesis pathway of ART.

It is well known that biosynthesis of ART includes three stages, that is, the formation of farnesyl pyrophosphate (FPP) from acetyl-CoA, the synthesis of sesquiterpene, and the formation of ART via lactonization (Figure 1). Farnesyl pyrophosphate synthase (FPS) catalyzes condensation of isopentenyl diphosphate (IPP) and dimethylallyl diphosphate (DMAPP) to produce FPP, which is the starting point of a large variety of important isoprenoid end products, including ART [9]. The cyclization of FPP to generate amorpha-4,11-diene catalyzed by amorpha-4,11-diene synthase (ADS) has been suggested as the first committed and limiting step in ART biosynthesis [10]. CYP71AV1, a cytochrome P_450_, could oxidize amorpha-4,11-diene to generate artemisinic aldehyde intermediates, which might be further catalyzed to dihydroartemisinic acid (DHAA) by artemisinic aldehyde Δ11(13) reductase (Dbr2) and aldehyde dehydrogenase (Aldh1) [11–13]. DHAA was supposed to be converted chemically into ART by an oxygen-mediated photochemical oxidation in vitro, which was a nonenzymatic process [14, 15]. Some experimental results also demonstrated that enzymatic catalysis or both enzymatic catalysis and autooxidation might occurr in the pathway from DHAA to ART in* A. annua *[16].

Feeding of precursors is one of the most effective strategies employed to increase the production of important secondary metabolites in cells and organ cultures [17]. Some precursors such as sodium acetate, mevalonic acid lactone, casein acid hydrolysate have been investigated to enhance yield of ART in plant and cell cultures of* A. annua *[17, 18]. However, little information is known about feeding DHAA, the immediate precursor of ART, to the plant cultured cells of* A. annua*. Does the feeding of DHAA increase the yield of ART? And what kind of mechanism exists in the procedures? At the current paper, effect of DHAA on the accumulation of ART in suspension-cultured cells of* A. annua* was investigated. The mechanism of biosynthesis pathway from DHAA to ART was also proposed.

## 2. Experimental Section 

### 2.1. General

^1^H and ^13^C nuclear magnetic resonance (NMR) was recorded on a Bruker DRX-400 spectrometer, the chemical shifts (*δ*) were given in ppm relative to TMS as an internal standard, and coupling constants were given in Hz. Silica gel (100–200 and 200–300 mesh) was used for column chromatography (CC), and silica GF_254_ (10–40*μ*) for TLC was supplied by the Qingdao Marine Chemical Factory, China. ODS (YMC Co., LTD, Japan) and Sephadex LH-20 (Pharmacia Co.) were also used for separation. HPLC analysis was performed on a Agilent 1200 liquid chromatography system (Palo Alto, CA, USA), equipped with vacuum degasser, quaternary gradient pump, autosampler, and a Alltech ELSD 2000ES (Grace Davison Discovery Sciences). An Agilent Hypersil ODS column (*φ*  4.6 mm × 250 mm, 5 *μ*m) and guard column (4.6 mm × 12.5 mm, 5 *μ*m) were used. A binary gradient elution system consisted of water (A) and methanol (B) and a separation procedure was achieved using the following gradient program: 0–5 min 40–50% B; 5–10 min 50–60% B; 10–15 min 60–70% B; 15–20 min 70–85% B; 20–25 min 100% B, and finally, reconditioning the column with 40% B isocratic for 2 min. The flow rate was 0.8 mL/min, and the system operated at 30°C. The detection wavelength was set at 230 nm. GC-MS was performed in a gas chromatographer GC7890-5975MSD (Agilent). The GC was set at the following conditions: the column: DB-5 (30 m × 0.25 mm, 0.25 *μ*m), the carrier gas: helium, pressure: 11.4 psi, total flow: 27.8 mL/min, column flow: 1.0 mL/min, purge flow: 3.0 mL/min, and a split ratio of 20 : 1. Column temperature was set at 80°C for 1 min, then 4°C/min to 206°C, 3°C/min to 230°C, and finally 15°C/min to 300°C for 20 min. Inlet heater was set at 280°C, solvent delay time was 4 min, and Mass scan parameter was from 35 to 500. The temperature of ms source is 230°C; ms quad was 150°C.

### 2.2. Chemicals

DHAA was extracted and isolated from* A. annua *by authors according to the referenced protocol [19]. The structure of DHAA was determined by MS and NMR. Its purity was > 98% by HPLC analysis. ART was purchased from Chengdu Mansite Biotechnology CO., LTD. Data for DHAA were shown as follow.

#### 2.2.1. DHAA

Colorless needle crystals (CDCl_3_). (*R*_*t*_ 26.81 min in GC-MS). ^1^H-NMR (*δ*, CDCl_3_) ppm: 5.14 (1H, s), 2.52 (2H, m), 2.0~1.9 (2H, m), 1.83 (1H, dd), 1.66 (3H, s), 1.21 (3H, d, *J* = 6.8 Hz), 1.14 (1H, dddd, *J* = 13.2, 12.4, 12.8, and 3.2 Hz), 0.99 (1H, dddd, *J* = 12.8, 12.0, 12.8, and 3.2 Hz), and 0.89 (3H, d, *J* = 6.8 Hz); ^13^C-NMR (*δ*, CDCl_3_) ppm: 41.7 (C-1), 25.7 (C-2), 26.6 (C-3), 135.9 (C-4), 119.3 (C-5), 36.3 (C-6), 43.5 (C-7), 27.4 (C-8), 35.2 (C-9), 27.6 (C-10), 42.2 (C-11), 183.8 (C-12), 15.0 (C-13), 19.0 (C-14), and 23.7 (C-15); EI-MS: 236 (2), 162 (100), and 121 (25).

### 2.3. Plant Cultured Cells

*A. annua *cells were subcultured routinely every 3 weeks using MS medium containing 2,4-dichlorophenoxyacetic acid (2,4-D, 0.5 mg/L) and 6-benzylaminopurine (6-BA, 1 mg/L) and transplanted to 500 mL conical flask containing 200 mL of medium and then cultured on a rotary shaker (110 rpm) for 13 days at 25°C in the dark.

### 2.4. Detection of Secondary Metabolites after Feeding of DHAA

Three groups of experiments were taken out to check whether biosynthesis process occurred. Group I contained cultured cells and DHAA; group II was the first control experiment, consisting of cultured cells but without DHAA; group III was the second control experiment, in which only DHAA existed. The procedure of experiment group I was as follows: DHAA (5 mg) in ethanol (0.1 mL) was administered to the flask containing the suspended cells (precultured for 13 days) of* A. annua* and then cocultured at 25°C on a rotary shaker in the dark (110 rpm) for two days. After incubation, cells and medium were separated by filtration with suction. Filtered medium was extracted with EtOAc and concentrated to dryness (fraction 1, Fr. 1), with the medium further extracted with n-BuOH and then treated with above method to obtain fraction 2 (Fr. 2). The cells were extracted with MeOH for 12 h and sonicated for 20 min. The MeOH fraction was concentrated and partitioned between H_2_O and EtOAc and EtOAc solution was concentrated to dryness (Fr. 3); then further H_2_O solution was extracted with n-BuOH and then treated with above method to obtain fraction 4 (Fr. 4). Four fractions were analyzed by TLC, HPLC, and GC-MS. To group II, 0.1 mL of ethanol was added to the medium. For group III, DHAA (5 mg) in ethanol (0.1 mL) was administered to the medium. Extraction and analysis processes of the two control experiments were the same as those described above.

### 2.5. Biosynthesis of ART and Its Analogs

DHAA (110 mg) was dissolved in ethanol (1.1 mL), distributed among 22 conical flasks with 13-day-old cells, and incubated for additional 2 days. The culture and extraction conditions were as same as above described. Fr. 3 was further purified on column chromatography by silica gel, Sephadex LH-20, and ODS. The same procedure was repeated 5 times.

### 2.6. Investigation of the Optimal Conditions for ART Synthesis

Cultured cells of* A. annua *(10 g) were transferred to a 500 mL conical flask containing 200 mL medium and cultured by continuous shaking for 13 days at 25°C. DHAA (12.5, 25, 50 mg/L) was added to the suspension cultures and incubated at 25°C in a rotary shaker (110 rpm). At 1-day intervals, three of the flasks in each concentration group were taken out from the rotary shaker, and then the cells and medium were separated by filtration. The extraction and analysis procedures were the same as those described above. The yield of ART was calculated on the basis of the peak area from HPLC using calibration curves prepared by HPLC-ELSD and was expressed as micrograms per liter culture.

### 2.7. Expression of ART Biosynthetic Genes

For semiquantitative RT-PCR analysis, DHAA (25 mg/L) was added to the cultured cells, with ethanol as control. After two days of coculture, total RNAs were prepared with Trizol reagent (Invitrogen, USA) by the method of Wen [20]. After the RNA pellet was dissolved in 20*μ*L of DEPC-treated water, transcript levels of HMGR, FPS, ADS, CYP71AV1, and CPR genes in elicited and control cultures were measured by semiquantitative RT-PCR using PrimeScript One Step Kit Ver.2 (TaKaRa Biotechnology Co., Ltd., Japan) according to manufacturer's manual. Gene-specific sense and antisense primers for HMGR, FPS, ADS, CYP71AV1, and CPR genes were designed in accordance with literature [21]. The PCR products were loaded onto 1.5% agarose gel and then the results were compared. RT-PCR images were obtained and band intensities were quantified by Image J software. All samples were assayed in triplicates, and the mean expression values were calculated.

## 3. Results 

### 3.1. Detection of Biosynthesis Products of DHAA by GC-MS

**Figure 2:**
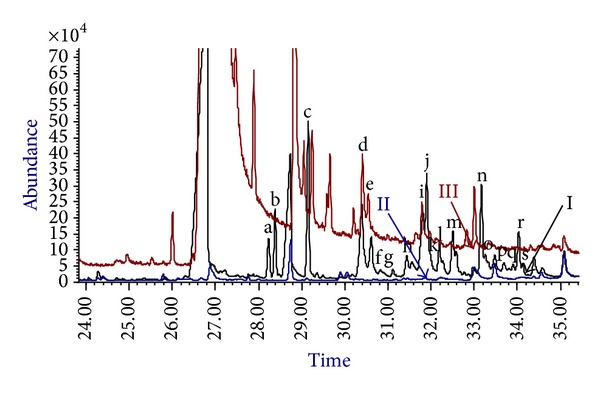
GC-MS detection of DHAA metabolites in suspension-cultured cells of* A. annua*. (I): suspension-cultured cells with DHAA; (II): suspension-cultured cells without DHAA; (III): media with DHAA but without suspension-cultured cells.

Biosynthesis products of DHAA were found in EtOAc extraction of group I (Figure 2). Compared the GC-MS spectra of group I and group II, at least 19 new peaks (a~s) appeared, which showed that enzyme-catalyzed reaction or autooxidation likely occurred in group I.

At least 16 new peaks (a~c, f~h, j~s) were generated from enzyme catalysis (Figure 2). Among them, 5 peaks were structurally elucidated to be dihydro-epi-deoxyarteannuin B (c), arteannuin I (f), arteannuin K (k), artemisinin (o), and 3-*β*-hydroxy-dihydro-epi-deoxyarteannuin B (r). Furthermore, the other 3 peaks were determined to be arteannuin H (j), arteannuin L (l), and arteannuin M (n) by GC-MS [16, 22]. Full scan mass spectra of peaks (j), (l), (n), and (o) were provided as supplementary materials. Results above showed that ART analogs were produced after the feeding of DHAA. Therefore, enzyme catalysis might be involved in the process.

### 3.2. Biosynthesis of ART and Its Analogs

**Figure 3:**
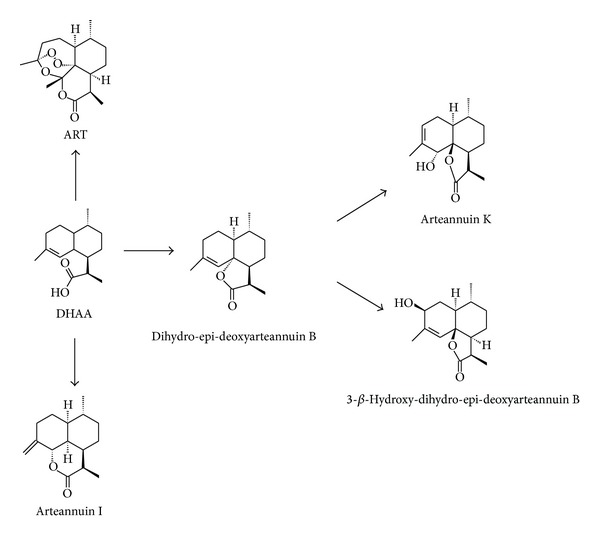
Proposed biosynthesis pathway of ART and its analogs from DHAA in suspension-cultured cells of* A. annua*.

Five biosynthesis products were isolated after DHAA was incubated with the suspension-cultured cells of* A. annua* for two days (Figure 3). Five compounds, ART, dihydro-epi-deoxyarteannuin B, arteannuin I, arteannuin K, and 3-*β*-hydroxy-dihydro-epi-deoxyarteannuin B, were elucidated by comparing the physicochemical properties and spectra data (NMR, EI-MS, and GC-MS) with those of references [21, 22]. Data were shown as follow.

#### 3.2.1. ART

Oil (*R*_*t*_ 33.256 min in GC-MS).^ 1^H-NMR (*δ*, CDCl_3_) ppm: 5.86 (1H, s), 3.40 (1H, m), 2.44 (1H, m), 2.04 (1H, m), 1.45 (3H, s, H-15), 1.38 (1H, m), 1.21 (3H, d, *J* = 6.8 Hz), and 1.00 (3H, d, *J* = 6.0 Hz); ^13^C-NMR (*δ*, CDCl_3_) ppm: 50.2 (C-1), 25.0 (C-2), 34.8 (C-3), 105.3 (C-4), 93.7 (C-5), 79.5 (C-6), 45.1 (C-7), 23.4 (C-8), 33.7 (C-9), 37.5 (C-10), 33.0 (C-11), 171.9 (C-12), 12.5 (C-13), 19.7 (C-14), and 25.2 (C-15); EI-MS: 282 (4), 250 (7), 195 (25), 179 (40), 166 (100), 151 (93), 137 (91), and 123 (15).

#### 3.2.2. Dihydro-epi-deoxyarteannuin B

Oil (*R*_*t*_ 29.142 min in GC-MS). ^1^H-NMR (*δ*, CDCl_3_) ppm: 5.64 (1H, s), 3.14 (1H, m), 2.15~2.05 (2H, m), 2.04 (1H, m), 1.89 (1H, m), 1.75~1.63 (3H, m), 1.69 (3H, s), 1.43 (1H, m), 1.20 (1H, m), 1.15 (3H, d, *J* = 7.2 Hz), 1.05 (1H, m), and 0.94 (3H, d, *J* = 6.4 Hz,); ^13^C-NMR (*δ*, CDCl_3_) ppm: 46.6 (C-1), 20.9 (C-2), 30.8 (C-3), 142.2 (C-4), 121.7 (C-5), 83.7 (C-6), 42.7 (C-7), 23.4 (C-8), 32.4 (C-9), 29.6 (C-10), 39.6 (C-11), 179.3 (C-12), 9.4 (C-13), 19.6 (C-14), and 23.7 (C-15); EI-MS: 234 (10), 190 (87), and 161 (100).

#### 3.2.3. Arteannuin I

Oil (*R*_*t*_ 30.833 min in GC-MS). ^1^H-NMR (*δ*, CDCl_3_) ppm: 5.09 (1H, s), 4.97 (1H, d, *J* = 11.5 Hz), 4.75 (1H, s), 2.80 (1H, m), 1.25 (3H, d,* J* = 7.2 Hz), and 0.92 (3H, d, *J* = 6.4 Hz); ^13^C-NMR (*δ*, CDCl_3_) ppm: 43.7 (C-1), 28.8 (C-2), 29.6 (C-3), 146.5 (C-4), 120.7 (C-5), 45.1 (C-6), 40.2 (C-7), 22.3 (C-8), 35.1 (C-9), 28.0 (C-10), 40.5 (C-11), 173.9 (C-12), 13.3 (C-13), 20.1 (C-14), and 107.3 (C-15); EI-MS: 234 (100), 206 (51), 191 (40), 177 (26), 123 (84), and 109 (27).

#### 3.2.4. Arteannuin K

Oil (*R*_*t*_ 31.885 min in GC-MS). ^1^H-NMR (*δ*, CDCl_3_) ppm: 5.56 (1H, d, *J* = 5.2 Hz), 3.77 (1H, d, *J* = 7.2 Hz), 3.16 (1H, m), 2.84 (3H, s), 2.76 (1H, m), 1.76 (3H, s), 1.06 (3H, d, *J* = 7.2 Hz), and 0.94 (3H, d, *J* = 6.4 Hz); ^13^C-NMR (*δ*, CDCl_3_) ppm: 38.4 (C-1), 27.1 (C-2), 124.0 (C-3), 132.3 (C-4), 69.2 (C-5), 84.9 (C-6), 37.9 (C-7), 24.3 (C-8), 32.0 (C-9), 32.9 (C-10), 38.4 (C-11), 178.2 (C-12), 8.7 (C-13), 19.2 (C-14), and 20.6 (C-15); EI-MS: 250 (1), 167 (100), 151 (20), 121 (11), and 84 (73).

#### 3.2.5. 3-*β*-Hydroxy-dihydro-epi-deoxyarteannuin B

Oil (*R*_*t*_ 33.777 min in GC-MS). ^1^H-NMR (*δ*, CDCl_3_) ppm: 5.81 (1H, s, *J* = 6.0 Hz), 3.92 (1H, d, *J* = 7.6), 3.26 (1H, m), 2.06 (1H, m), 1.79 (3H, s), 1.07 (3H, d, *J* = 7.2 Hz), and 0.95 (3H, d, *J* = 6.0 Hz); ^13^C-NMR (*δ*, CDCl_3_) ppm: 46.2 (C-1), 31.6 (C-2), 69.9 (C-3), 144.7 (C-4), 123.5 (C-5), 82.1 (C-6), 42.2 (C-7), 23.4 (C-8), 32.2 (C-9), 29.4 (C-10), 39.1 (C-11), 177.8 (C-12), 8.8 (C-13), 18.8 (C-14), and 18.6 (C-15); EI-MS: 250 (20), 177 (55), 166 (73), 151 (100), and 84 (38).

### 3.3. Optimal Biosynthesis Conditions of ART and Its Analogs

**Figure 4:**
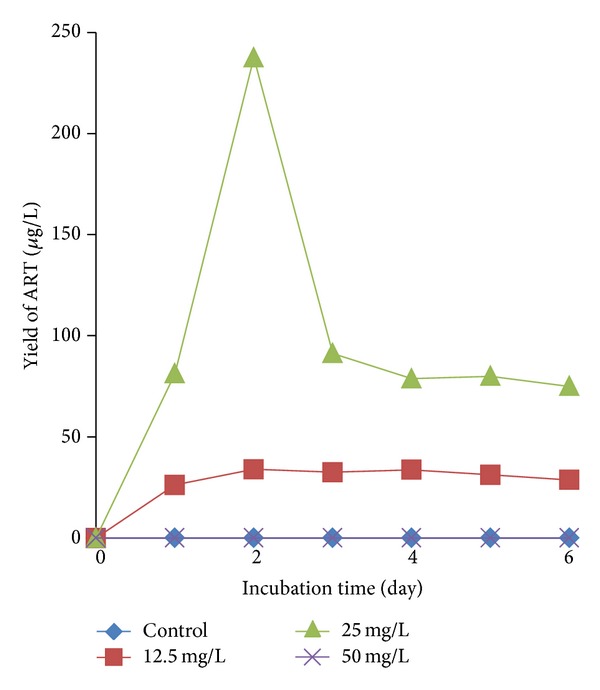
Effect of DHAA on the yield of ART.

To determine the optimal biosynthesis conditions, different concentrations of DHAA and the coculture time were investigated. The results indicated that there was no ART detected in the two control groups and DHAA (50 mg/L) treated group (Figure 4). High amount of DHAA (50 mg/L) showed great toxicity to the culture cells, which made the cells dead from the first day. The highest yield (237.5 *µ*g/L) occurred on day 2 after the coculture in DHAA (25 mg/L) treated group. Therefore, the optimal condition for ART production was shown as follow: precultured time for cells of* A. annua*: 13 days; concentration of DHAA: 25 mg/L; cocultured time: 2 days.

### 3.4. Expression Analysis of ART Biosynthetic Genes

**Figure 5:**
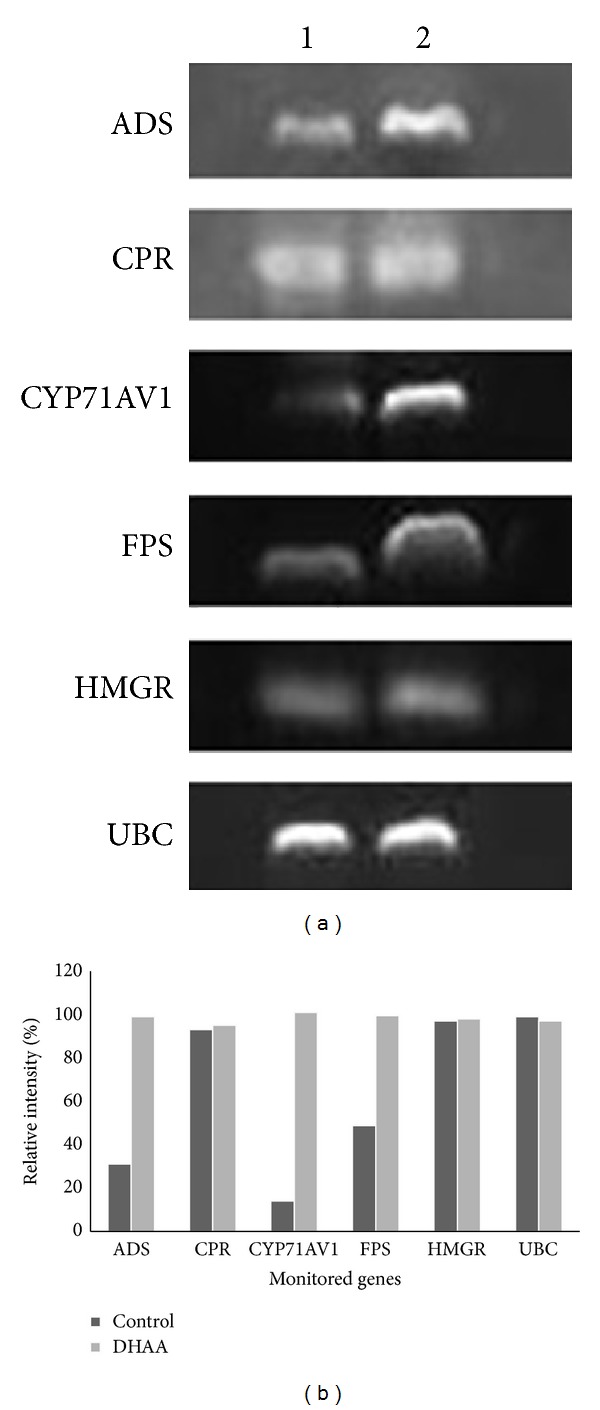
Effect of DHAA on the transcript levels of ADS, CPR, CYP71AV1, FPS, and HMGR genes in suspension-cultured cells of* A. annua*. (1): control group; (2): DHAA (25 mg/L) treated group, UBC was used as the internal standard. The band intensities were quantified by Image J software, and the relative expression was the ratio of intensity of control group to that of DHAA group.

As shown in Figure 5, ADS, CYP71AV1, and FPS transcript levels in cultured cells of* A. annua *were upregulated by the addition of DHAA, in which the transcript abundances were 3.19-, 7.21-, and 2.04-fold higher than those of the control group, respectively. While CPR and HMGR transcripts were detected both in the control group and in the DHAA-treated group, the transcript abundances for these two groups appeared little different.

## 4. Discussion

DHAA was found to undergo slow autooxidation to ART and some natural products in the presence of light and singlet oxygen. This process took several weeks and no transformations were observed when DHAA was kept in the dark [14]. In our experiment, ART and other products were obtained after two days administration of DHAA to the cultured cells of* A. annua*. This biosynthesis process occurred in the dark, and such a circumstance was not favorable for autooxidation. In contrast, ART and the other products were not found in the two control groups (Figure 2). The results indicated that in the biosynthesis process from DHAA to ART in suspension-cultured cells (group I), enzyme catalysis existed. This result is also in accordance with reference [21]. To verify above result, the experiment of ART biosynthetic genes expressions was designed in the present study.

RT-PCR analysis of five important genes in the biosynthetic pathway of ART, namely, HMGR, FPS, ADS, CYP71AV1, and CPR, was carried out under the optimal condition for ART production. Under this condition, the content of ART was the highest (237.5 *µ*g/L). Results indicated that DHAA was the essential intermediate for the biosynthesis of ART and it could greatly increase the expression levels of ADS, CYP71AV1, and FPS genes. It was reported that the content of ART would increase when ADS and CYP71AV1 were induced [23]. The results of our study showed that the biosynthesis of ART was enzymatic regulation and the upregulation of genes leading to the enhancement in production of ART.

In this study, the suspension cells of* A. annua* were cultured on MS medium, with 0.5 mg/L of 2,4-dichlorophenoxyacetic acid (2,4-D) and 1 mg/L of 6-benzylaminopurine (6-BA) at 25°C in the dark. The culture condition was different from the reference in which high concentration of ART was reported [16]. The main difference between the culture conditions was the usage of hormones. Naphthylacetic acid (NAA, 2 mg/L) was used in the reference while 2,4-D and 6-BA were used in our study. It is well known that hormones play an important role in the production of secondary metabolites. Different hormones may result in different type of metabolites with different concentration. An example in case was that 2,4-D inhibited alkaloid accumulation in the cultured cells of* Catharanthus roseus *[24]. More interesting, genes were repressed in suspension-cultured cells of* C. roseus *with 2,4-D [25].

Another possible reason for the low yield of ART in our investigation was that the accumulation of some genes expression products might be light related [26]. In our experiment, research was carried out in the dark. Therefore, transcript abundance of those genes may be blocked. Furthermore, the addition of DHAA could effectively enhance the expression of three important genes, FPS, ADS, and CYP71AV1, leading to the enhancement of ARTs in suspension-cultured cells of* A. annua*.

To our knowledge, this is the first report to investigate the enzymatic synthesis of ART and its four analogs from DHAA in suspension-cultured cells of* A. annua*. The yields of ARTs were significantly increased after DHAA was fed to the suspension-cultured cells of* A. annua*. Positive correlation was observed between ART content and the upregulation of FPS, ADS, and CYP71AV1 genes.

## References

[1] López C, Saravia C, Gomez A, Hoebeke J, Patarroyo MA (2010). Mechanisms of genetically-based resistance to malaria. *Gene*.

[2] Blazquez AG, Fernandez-Dolon M, Sanchez-Vicente L (2013). Novel artemisinin derivatives with potential usefulness against liver/colon cancer and viral hepatitis. *Bioorganic and Medicinal Chemistry*.

[3] Brown GD (2010). The biosynthesis of artemisinin (Qinghaosu) and the phytochemistry of *Artemisia annua* L. (Qinghao). *Molecules*.

[4] Paddon CJ, Westfall PJ, Pitera DJ (2013). High-level semi-synthetic production of the potent antimalarial artemisinin. *Nature*.

[5] Kjær A, Verstappen F, Bouwmeester H (2013). Artemisinin production and precursor ratio in full grown *Artemisia annua* L. plants subjected to external stress. *Planta*.

[6] Ro D, Paradise EM, Quellet M (2006). Production of the antimalarial drug precursor artemisinic acid in engineered yeast. *Nature*.

[7] Covello PS (2008). Making artemisinin. *Phytochemistry*.

[8] Graham LA, Besser K, Blumer S (2010). The genetic map of Artemisia annua L identifies loci affecting yield of the antimalarial drug artemisinin. *Science*.

[9] Newman JD, Chappell J (1999). Isoprenoid biosynthesis in plants: carbon partitioning within the cytoplasmic pathway. *Critical Reviews in Biochemistry and Molecular Biology*.

[10] Bouwmeester HJ, Wallaart TE, Janssen MHA (1999). Amorpha-4,11-diene synthase catalyses the first probable step in artemisinin biosynthesis. *Phytochemistry*.

[11] Teoh KH, Polichuk DR, Reed DW, Nowak G, Covello PS (2006). *Artemisia annua* L. (Asteraceae) trichome-specific cDNAs reveal CYP71AV1, a cytochrome P450 with a key role in the biosynthesis of the antimalarial sesquiterpene lactone artemisinin. *FEBS Letters*.

[12] Zhang Y, Teoh KH, Reed DW (2008). The molecular cloning of artemisinic aldehyde ∆11(13) reductase and its role in glandular trichome-dependent biosynthesis of artemisinin in *Artemisia annua*. *The Journal of Biological Chemistry*.

[13] Teoh KH, Polichuk DR, Reed DW, Covello PS (2009). Molecular cloning of an aldehyde dehydrogenase implicated in artemisinin biosynthesis in *Artemisia annua*. *Botany*.

[14] Sy L, Brown GD (2002). The mechanism of the spontaneous autoxidation of dihydroartemisinic acid. *Tetrahedron*.

[15] Schramek N, Wang H, Römisch-Margl W (2010). Artemisinin biosynthesis in growing plants of *Artemisia annua*. A ^13^CO_2_ study. *Phytochemistry*.

[16] Brown GD, Sy LK (2004). *In vivo* transformations of dihydroartemisinic acid in *Artemisia annua plants*. *Tetrahedron*.

[17] Baldi A, Dixit VK (2008). Yield enhancement strategies for artemisinin production by suspension cultures of *Artemisia annua*. *Bioresource Technology*.

[18] Zeng Y, Yan F, Tang L, Chen F (2003). Increased crocin production and induction frequency of stigma-like-structure from floral organs of *Crocus sativus* by precursor feeding. *Plant Cell, Tissue and Organ Culture*.

[19] Wallaart TE, Van Uden W, Lubberink HGM, Woerdenbag HJ, Pras N, Quax WJ (1999). Isolation and identification of dihydroartemisinic acid from *Artemisia annua* and its possible role in the biosynthesis of artemisinin. *Journal of Natural Products*.

[20] Wen W, Zhu JH, Liu JW, Yu RM (2012). Impact of artemisinic acid on the growth and catharanthine production in *Catharanthus roseus* cell culture. *Journal of Medicinal Plants Research*.

[21] Jing F, Zhang L, Li M (2009). Abscisic acid (ABA) treatment increases artemisinin content in *Artemisia annua* by enhancing the expression of genes in artemisinin biosynthetic pathway. *Biologia*.

[22] Sy LK, Brown GD, Haynes R (1998). A novel endoperoxide and related sesquiterpenes from *Artemisia annua* which are possibly derived from allylic hydroperoxides. *Tetrahedron*.

[23] Yu ZX, Li JX, Yang CQ, Hu WL, Wang LJ, Chen XY (2012). The jasmonate-responsive AP2/ERF transcription factors AaERF1 and AaERF2 positively regulate artemisinin biosynthesis in *Artemisia annua* L.. *Molecular Plant*.

[24] Arvy MP, Imbault N, Naudascher F, Thiersault M, Doireau P (1994). 2,4-D and alkaloid accumulation in periwinkle cell suspensions. *Biochimie*.

[25] Hedhili S, Courdavault V, Giglioli-Guivarc'h N, Gantet P (2007). Regulation of the terpene moiety biosynthesis of *Catharanthus roseus* terpene indole alkaloids. *Phytochemistry Reviews*.

[26] Hong G, Hu W, Li J, Chen X, Wang L (2009). Increased accumulation of artemisinin and anthocyanins in *Artemisia annua* expressing the arabidopsis blue light receptor CRY1. *Plant Molecular Biology Reporter*.

